# Diagnostic performance and factors influencing the accuracy of EUS-FNA of pancreatic neuroendocrine neoplasms

**DOI:** 10.1007/s00535-016-1164-6

**Published:** 2016-01-14

**Authors:** Susumu Hijioka, Kazuo Hara, Nobumasa Mizuno, Hiroshi Imaoka, Vikram Bhatia, Mohamed A. Mekky, Kenichi Yoshimura, Tsukasa Yoshida, Nozomi Okuno, Nobuhiro Hieda, Masahiro Tajika, Tsutomu Tanaka, Makoto Ishihara, Yasushi Yatabe, Yasuhiro Shimizu, Yasumasa Niwa, Kenji Yamao

**Affiliations:** 1 Department of Gastroenterology, Aichi Cancer Center Hospital, 1-1 Kanokoden, Chikusa-ku, Nagoya, 464-8681 Japan; 2 Department of Gastroenterology, Fortis Escorts Liver and Digestive Institute, New Delhi, India; 3 Department of Tropical Medicine and Gastroenterology, Assiut University Hospital, Assiut, Egypt; 4 Innovative Clinical Research Center, Kanazawa University, Kanazawa, Japan; 5 Department of Endoscopy, Aichi Cancer Center Hospital, Nagoya, Japan; 6 Department of Pathology and Molecular Diagnostics, Aichi Cancer Center Hospital, Nagoya, Japan; 7 Department of Surgery, Aichi Cancer Center Hospital, Nagoya, Japan

**Keywords:** EUS-FNA, Pancreatic neuroendocrine neoplasms, Diagnosability

## Abstract

**Background:**

Multiple studies have investigated sampling adequacy of endoscopic ultrasound-guided fine needle aspiration (EUS-FNA) for pancreatic neuroendocrine neoplasms (pNENs). However, none have described the diagnostic performance of EUS-FNA for pNENs, or the influencing factors. The aim of this study was to evaluate the diagnostic accuracy of EUS-FNA, with post-operative pathological diagnosis as the gold standard, and factors predictive of inadequate EUS sampling.

**Methods:**

From 1998 to 2014, a total of 698 patients underwent pancreatic resection and 1455 patients underwent EUS-FNA sampling for pancreatic lesions. A total of 410 cases underwent both surgical resection and preceding EUS-FNA. Of these, 60 cases (49 true pNEN, nine non-diagnostic, two misdiagnoses) were included. We studied diagnostic performance of EUS-FNA and factors that were associated with failed diagnosis.

**Results:**

Of the 60 cases, EUS-FNA yield was 49 true-positive cases, two misdiagnoses, and nine non-diagnostic cases (including six suggestive cases). Sensitivity, specificity, and accuracy were 84.5, 99.4, and 97.3 %, respectively; including the six suggestive cases, diagnostic values were 94.8 % sensitivity (55/58), 99.4 % specificity (350/352), and 98.7 % accuracy (405/410). In multivariate analysis, sampling adequacy rates were significantly lower when lesions were located in the pancreatic head [odds ratio (OR) = 10.0] and in tumor-rich stromal fibrosis (OR = 10.45). Tumor size, needle type, tumor grading, presence of cystic component, and time period were not significant factors.

**Conclusions:**

EUS-FNA offers high accuracy for pNEN. However, location of the tumor in the pancreatic head and presence of rich stromal fibrosis negatively impacts sampling adequacy.

**Electronic supplementary material:**

The online version of this article (doi:10.1007/s00535-016-1164-6) contains supplementary material, which is available to authorized users.

## Introduction

Pancreatic neuroendocrine neoplasms (pNENs) are rare pancreatic tumors, estimated to comprise 2–3 % of all pancreatic neoplasms [1]. Recent progress in cross-sectional imaging has resulted in a substantial rise in detection rates for pNEN, even when small and asymptomatic. However, histological evidence is mandatory in addition to suggestive imaging. Endoscopic ultrasound-guided fine needle aspiration (EUS-FNA) is now accepted as the primary sampling technique for pancreatic tumors [2, 3], with 83.3–93 % sampling adequacy rates [4–7]. The 2010 revised World Health Organization classification grades pNEN as NET-G1 G2 and NEC, based on Ki67 staining or mitosis rates [8]. Concordance rates between grading of pNENs by EUS-FNA and postoperative histology are reportedly within the range of 77–89.5 % [9–14]. We have previously reported concordance rates as high as 90 % when EUS-FNA samples contain more than 2000 tumor cells [11].

Most previous reports describing EUS-FNA sampling of pNENs have only focused on sampling adequacy rates, rather than diagnostic accuracy. In addition, no studies have investigated factors related to sampling adequacy for pNENs. The present study, therefore, estimated the EUS-FNA diagnostic accuracy rates in cases of surgically confirmed pNEN and examined various factors related to sampling inadequacy.

## Materials and methods

### Study design and patient selection

We retrospectively reviewed the data registry of all patients with pancreatic neoplasm who underwent surgical resection preceded by EUS-FNA at Aichi Cancer Center, Nagoya, Japan, between 1998 and 2014.

**Fig. 1:**
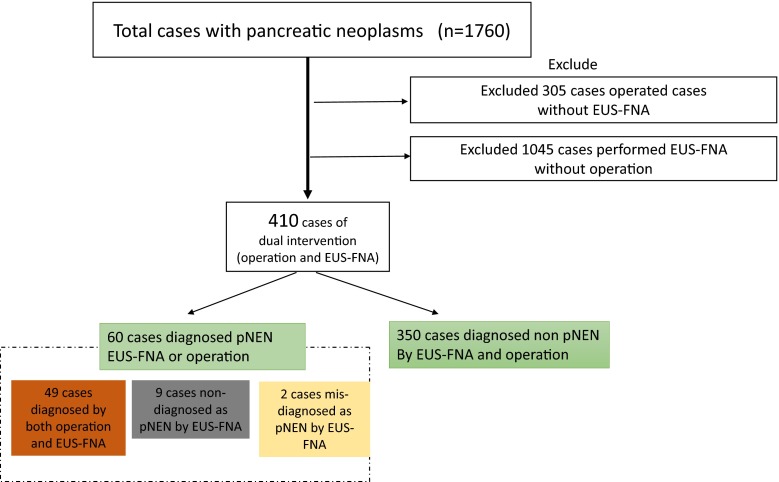
Algorithm for patient inclusion and exclusion

A total of 698 cases underwent pancreatic resection (including 74 cases with pNEN), 1455 cases underwent EUS-FNA of pancreatic solid masses (including 89 cases with pNEN), and 410 cases underwent both EUS-FNA sampling and surgical resection of the pancreatic neoplasms. Of the cases with dual intervention, 60 cases were included in this study. Forty-nine of these cases were correctly diagnosed by EUS-FNA preoperatively, two cases were misdiagnosed as pNEN by EUS-FNA, and nine surgically confirmed cases of pNEN were not diagnosed by preoperative EUS-FNA. Figure 1 summarizes the patient selection criteria.

This study was approved by the institutional review board of our institution.

### EUS-FNA procedures

EUS-FNA was performed using a GF-UC30P (1998-2001), GF-UC240P-AL5 (2001-present), or GF-UCT260-AL5 (2011-present) convex array echoendoscope (Olympus Corporation, Tokyo, Japan) connected to an ultrasound scanning system (Envision Plus; Dornier MedTech, Munich, Germany or SSD-5500, Prosound SSD α-5,10; Hitachi Aloka Medical, Tokyo, Japan), as appropriate according to a previously described methodology [11, 15]. Different types of needles (19-, 22-, or 25G Echo Tip Ultra; Cook Medical, IN, USA, or NA-200H-8022; Olympus Corporation or Expect; Boston Scientific, Tokyo, Japan) were employed for the sampling. The type and size of needle were chosen at the discretion of the endosonographer. We uniformly used negative suction with a 10-mL or 20-mL syringe during all FNA procedures. EUS-FNA was performed by five expert endosonographers (K.Y., K.H., N.M., H.I., S.H.) or under their direct supervision.

### Cytology and immunohistological diagnosis

After spraying the aspirated material onto glass slides, one slide was fixed by air-drying, stained with modified Giemsa stain (Diff-Quik; Kokusai Shiyaku, Kobe, Japan), and reviewed immediately (on-site cytopathological evaluation) by the cytopathologist or cytotechnologist to ensure specimen adequacy. Another slide was fixed by immediate immersion in 95 % alcohol and then stained with Papanicolaou stain. Additional material was obtained from each lesion unless on-site evaluation confirmed the presence of malignant cells or a sufficient number of representative cells from the lesion. Subsequently, the remaining material, as well as the specimen obtained by one more pass, was submitted for cell-block preparation. The cell-block material was processed by fixation in 10 % neutral-buffered formalin solution, then embedded in paraffin to be handled as a routine tissue block. Thin sections from paraffin-embedded cell blocks were cut and stained with hematoxylin and eosin (HE). All diagnoses were confirmed by a combination of characteristic HE features and immunohistochemistry (IHC) showing expression of chromogranin A and/or synaptophysin. In this study, sampling adequacy rate was defined by the proportion of lesions in which adequate material for cytopathological diagnoses could be obtained. Ki67 labeling index (LI) was used for tumor grading. Mitotic count was not performed on our cellblock specimens, because at least 50 high-power fields are required for reliable estimation [8, 16], a requirement that could not be fulfilled in most samples.

### Study definitions

#### EUS-FNA diagnostic accuracy

EUS-FNA diagnosis of pNEN was considered “accurate” when the cell block, including IHC staining results, matched the final diagnosis. In addition, when the cell block including IHC diagnoses was reported as “suspicious” or “consistent”, we included them as accurate diagnoses for pNEN. When cytology and cell-block and/or IHC diagnoses were reported as “suspicious for pNEN”, we included these as “suggestive” for pNEN. When cytology cell-block diagnoses were reported as “atypical” and inadequate for IHC, we included these as “inaccurate diagnoses” for pNEN. Non-diagnostic included “suggestive” and “inaccurate diagnoses”. The criterion standard for “final diagnosis” was the surgical histopathological results for resected specimens alone.

#### Grading of stromal fibrosis

We evaluated the degree of stromal fibrosis using the maximal section of the resected specimens. We defined “rich fibrosis” when stromal fibrosis occupied more than 30 % of the total tumor area [17].

### Magnetic resonance imaging (MRI) for evaluation of fibrosis

**Fig. 2:**
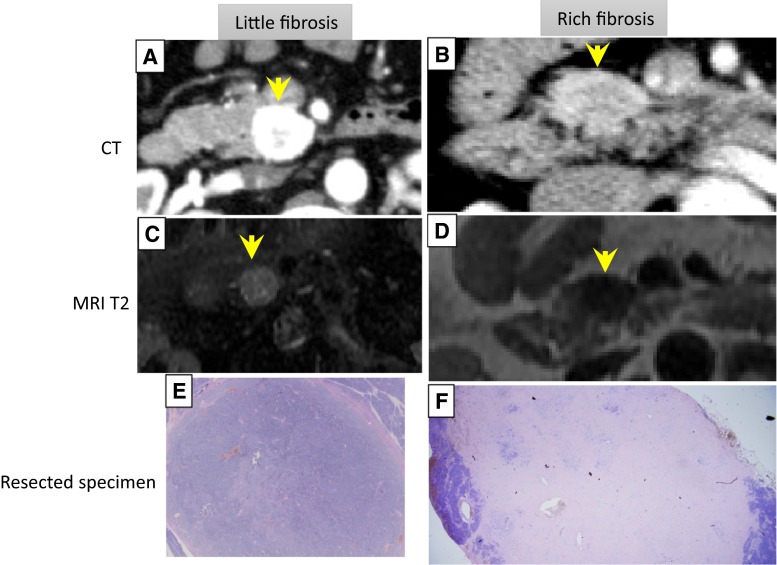
Representative cases of little and rich stromal fibrosis. **a**, **c**, **e** Images of pNEN with little fibrosis. **b**, **d**, **f** Images of pNEN with rich fibrosis. **a**, **b** CT shows strong and moderate hypervascularity in the tumor (*arrow*). **c**, **d** T2-weighted imaging shows a hyper- and hypointense mass in the pancreas head (*arrow*). **e**, **f** Low-power image showing weak and numerous fibrosis

The quantity of fibrosis was evaluated by T2-weighted imaging (WI). Cell-rich tumors were depicted as high-intensity on T2-WI [18], while tumors with more fibrosis were depicted as iso- or low-intensity (Fig. 2). The definition of signal intensity (hyper/iso/hypo) was the result compared with the surrounding parenchyma on T2-WI. The MRI findings were interpreted by mutual discussion between one gastroenterologist (S.H.) and one radiologist (Y.S.). For tumors showing cystic degeneration, we evaluated the MRI findings in the recognizable solid components of the tumor.

### Factors affecting accuracy of EUS-FNA for pNEN

Factors affecting the accuracy of EUS-FNA were analyzed using uni- and multivariate analyses. Variables employed for univariate analyses were location of the lesion (pancreatic head, body/tail), size of the lesion (≤10, 10–20 mm, >20 mm), needle size (19G vs. 22G vs. 25G), presence or absence of cystic degeneration, grading of malignancy (G1 or G2/NEC), grading of fibrosis (<30 vs. ≥30 %), and period during which EUS-FNA procedure was performed (period I: 1998–2008, comprising the first 30 cases; period II: 2009–2014, comprising the remaining 28 cases). As for needle size, five cases underwent EUS-FNA using two types of needles (22- and 25G needles). These five cases were thus excluded, and the remaining 53 cases were analyzed (19G, *n* = 3; 22G, *n* = 46; 25G, *n* = 5).

### Statistical analysis

We used the Chi squared test for univariate analyses, and logistic regression analysis for multivariate analysis. Values of *P* < 0.05 were regarded as statistically significant. All statistical analyses were undertaken using SPSS version 22 software (IBM, Tokyo, Japan).

## Results

Of 410 cases with dual EUS-FNA and surgical resection for a given pancreatic lesion, a total of 60 cases (51 % women; mean age, 55.7 ± 14.1 years) who fulfilled our inclusion criteria were identified.

Mean tumor size was 24.1 ± 21.3 mm (range 5–130 mm). Twenty-three tumors (38.3 %) were located in the head and 37 (61.6 %) in the body and tail. Nineteen lesions (31.6 %) displayed a cystic component. Eight patients (13.3 %) had liver metastasis. In terms of grading, 58 pNENs were classified as G1, G2, and NEC in 33 (55.0 %), 22 (36.6 %), and three cases (5.0 %), respectively. A definitive diagnosis and grading by surgical resection were achieved in all cases. Table 1 summarizes these characteristics.

**Table 1:** Characteristics of the 60 tumors (58 pNEN, 2 non-pNEN) *n* = 60

Size (mm)	
Mean ± SD	24.1 ± 21.3 mm
Location	
Head	23 (38.3 %)
Body	25 (41.6 %)
Tail	12 (20 %)
Cystic component	
Yes	19 (31.6 %)
No	41 (68.3 %)
Distant metastasis	
Yes	8 (13.3 %)
No	52 (86.6 %)
Grading	
G1	33 (55.0 %)
G2	22 (36.6 %)
NEC	3 (5.0 %)
Non-pNEN	2 (3.3 %)

*pNEN* pancreatic neuroendocrine neoplasm

### Diagnostic yield of EUS-FNA for pNENs

Of the 60 cases, the EUS-FNA diagnosis was classified as non-diagnostic, misdiagnosis, and diagnostic in nine (15.0 %), two (3.3 %), and 49 cases (81.6 %), respectively. In three of nine non-diagnostic cases, because of an insufficient specimen, suitable evaluation of IHC (chromogranin A and/or synaptophysin) could not be performed. However, in the remaining six cases, a diagnosis of pNEN was suspected based on HE staining and/or IHC. The two misdiagnosed tumors were paraganglioma and solid-pseudopapillary neoplasm (SPN) (Table 2). The paraganglioma was misdiagnosed as NET-G2 because the tumor cells were relatively uniform in size and shape, with round nuclei showing slight atypia, with finely dispersed chromatin. IHC staining yielded positive results for chromogranin A and synaptophysin, and negative results for cytokeratin7 and CDX2. Ki67 LI was estimated at 10 % (Figure S3). SPN was misdiagnosed as NET-G1 because slightly atypical cells with relatively uniform shape and agglomeration without pseudopapillary structures were seen. IHC staining of chromogranin A and synaptophysin were positive (chromogranin A was focally positive), cytokeratin7 and CDX2 were negative, and Ki67LI was estimated as 1 %. IHC for β-catenin was not performed because the results of HE staining, chromogranin A and synaptophysin staining corresponded for pNEN (Figure S4). The remaining 49 cases were diagnosed as pNEN by EUS-FNA and confirmed after surgery. In the TN group that included 350 cases, there was no cases with insufficient material by EUS-FNA. The diagnostic yield of EUS-FNA was: sensitivity, 84.5 % (49/58); specificity, 99.4 % (350/352); and accuracy, 97.3 % (399/410). Including the six “suggestive” cases as diagnostic, sensitivity was 94.8 % (55/58), specificity was 99.4 % (350/352), and accuracy was 98.7 % (405/410). Details of the diagnostic performance are shown in Table 3.

**Table 2:** Detail characteristics of two misdiagnosed cases

Case	Final diagnosis	Age (years)/Sex	Location	Size (mm)	Contrast-enhanced CT	Calcification	Cystic change	EUS-FNA needle size	CGA^a^/SYP^b^
1	SPN	32/M	Head	20	Hypovascular	+	−	22G	+(focal)/+
2	Paraganglioma	48/F	Body	30	Hypervascular	−	+	22G	+/+

^a^Chromogranin A

^b^Synaptophysin

**Table 3:** Diagnostic yield of EUS-FNA for pNEN (total cases of dual intervention, *n* = 410)

		Operation	
		PNEN	Non-pNEN
EUS-FNA	pNEN	49 (TP)	2 (FP)
	Non-pNEN^a^	9 (FN)	350 (TN)

EUS-FNA was classified as non-diagnostic in nine cases, misdiagnosis in two cases, and diagnostic in 49 cases

*pNEN* pancreatic neuroendocrine neoplasm, *EUS-FNA* endoscopic ultrasound-guided fine needle aspiration, *TN* true negative, *FN* false negative, *FP* false positive, *TP* true positive

^a^Included insufficient material

### Factors related to sampling adequacy

To clarify factors affecting the sampling adequacy of EUS-FNA for pNEN, uni- and multivariate analyses were conducted (Table 4). Both uni- and multivariate analyses revealed that tumor location and quantity of stromal fibrosis were significant independent factors affecting sampling adequacy. Lesions that were located in the pancreatic body or tail showed higher sampling adequacy rates than lesions located in the pancreatic head [*P* = 0.04; odds ratio (OR) = 10.0]. Sampling adequacy was lower when the tumor included rich stromal fibrosis (*P* = 0.03; OR = 10.45). On the other hand, tumor size, type of needle, grading, presence of cystic component, and study period were not found to be significant factors.

**Table 4:** Uni- and multivariate analyses of factors affecting sampling adequacy (58 pNEN)

Variable	Number	Accuracy (%)	Univariate analysis *P*	Multivariate analysis *P*	Odds ratio
Location					
Head	23	69.6	0.02	0.04	10.0
Body/tail	35	94.3			
Needle^a^					
19G	3	66.7	0.08		
22G	46	95.6			
25G	4	75.0			
Tumor size (mm)					
<10	15	100	0.13		
10–20	18	83.3			
>20	25	76.0			
Cystic component					
Present	17	76.5	0.24		
Absent	41	87.8			
Grading					
G1	33	87.9	0.32		
G2 or NEC	25	80.0			
Stromal fibrosis (%)					
<30	42	92.9	0.01	0.03	10.45
>30	16	62.5			
Period					
1998–2008	30	80.0	0.47		
2009–2014	28	89.3			

^a^Five patients in whom more than one needle was used were excluded

### Relationships between T2-weighted images and stromal fibrosis in pNEN

Of the 58 resected PNENs, 30 cases (51.7 %) had undergone MRI preoperatively. Cases showing rich fibrosis (>30 % fibrosis) were more often (*P* < 0.001) seen as iso- or low-intensity lesions on T2-WI (Table 5). Iso- or lowintensity appearance of pNENs on T2-WI had 100 % sensitivity, 81.8 % specificity, and 96.6 % diagnostic accuracy for the presence of rich fibrosis.

**Table 5:** Relationship between T2-weighted imaging and stromal fibrosis in pNEN (30 cases)

	Stromal fibrosis	
	Little	Rich
MRI-T2 WI		
Low-iso intensity	4 (13.3 %)	8 (26.6 %)
High intensity	18 (60 %)	0 (0 %)

## Discussion

A number of reports have described the excellent diagnostic ability of EUS-FNA for pNEN, with sensitivity of 83.3–93 % [4–7]. EUS-FNA is imperative for preoperative diagnosis of pNEN. However, around 10–15 % of cases remain undiagnosed despite EUS-FNA. No previous reports have discussed factors related to inadequate sampling of pNENs by EUS-FNA. Additionally, previous reports have only described sensitivity, without an estimate of true-negative and false-positive cases. This is the first report to calculate the sensitivity, specificity, and accuracy of EUS-FNA for pNENs and to investigate factors affecting the sampling adequacy of EUS-FNA. Surprisingly, we found that tumor size was not a significant predictor of sampling adequacy. In fact, 15 cases (25 %) had tumor size <10 mm (4–10 mm), and these could all be diagnosed by EUS-FNA. The reason for such high yield may be the high cellularity and minimal stromal fibrosis in small tumors. On the other hand, we found that tumor location and the amount of intra-tumoral fibrosis were independent predictors of sampling adequacy. Some previous reports have reached similar conclusions about the influence of tumor location on the diagnostic yield of EUS-FNA [15, 19]. Tumors with rich stromal fibrosis (>30 %) have a lower diagnostic yield on EUS-FNA, compared with tumors with minimal fibrosis (OR = 10.45; *P* = 0.03). Intra-tumoral fibrosis has been postulated to result from local serotonin production [17, 20, 21], as serotonin has been implicated in fibrogenesis. Carcinoid tumors of the midgut, in which serotonin is the predominant hormone secreted by neoplastic cells, are usually associated with extensive fibrosis [21]. In addition, serotonin has been shown to stimulate fibroblast mitosis in cell cultures [22]. In our cases, IHC for serotonin was not carried out, and whether the fibrosis correlated with local serotonin production remains speculative. How can we improve the diagnosability of pNEN with abundant fibrosis? The addition of T2-WI, which vividly depicts the quantity of fibrosis, will improve the diagnostic yield of pNEN. Most pNENs are hyperintense on T2-WI, but pNEN with abundant stromal fibrosis appears iso- or hypointense [23, 24]. In our series, 81.8 % (18/22) of cases with minimal stromal fibrosis showed hyperintensity on T2-WI, whereas 100 % (8/8) of cases with rich stromal fibrosis were iso- or hypointense on T2-WI (*P* < 0.001). We, therefore, recommend not only contrast-enhanced CT, but also MRI without contrast if pNEN is suspected. If an iso- or hypointense lesion is found on T2-WI, pNEN with rich fibrosis should be suspected. In such cases, particular attention must be paid to obtaining adequate tissue during EUS-FNA. Contrast-enhanced EUS (CE-EUS) may represent an attractive option in such cases. CE-EUS plays an important role in finding a specific site within a lesion that would be more suitable for EUS-FNA. Identification of hypervascular sites in such lesions may help avoid sampling rich fibrous areas [25]. Other options are to use high negative-pressure suction techniques in EUS-FNA [26] or a thicker needle [6].

We encountered two false-positive results for pNEN. The final diagnoses in these cases were paraganglioma and SPN. A report by Kari et al. [27] showed that 80 % of lesions misclassified as pNEN were actually SPN. Usually, FNA samples demonstrate a pseudopapillary pattern with fibrovascular stalks in SPN. However, in some cases with material crushed during aspiration or inadequate sampling, characteristic features of SPN may not be evident. Additionally, chromogranin A and/or synaptophysin staining is sometimes positive in SPN [28]. Indeed, our case of SPN did not show the classic features such as pseudopapillary pattern with fibrovascular stalks, and positive staining results were obtained for both chromogranin A and synaptophysin. Staining for β-catenin, E-cadherin, and CD10 may be able to better distinguish between pNEN and SPN [29], particularly using the nuclear staining distribution for β-catenin. Therefore, in cases of suspected SPN, these specialized IHC panels may be required.

The second case misdiagnosed as pNEN actually represented paraganglioma. This patient was asymptomatic before and during EUS-FNA, and even on retrospective review of CT images, the location of the tumor was difficult to identify as retroperitoneal. A case of similar misdiagnosis has been reported [30]. In the case of paraganglioma, EUS-FNA is relatively contraindicated because it may cause a severe hypertensive crisis during EUS-FNA [31]. Most paragangliomas show cystic degeneration, as in our case. When paraganglioma is suspected, meta-iodobenzylguanidine (MIBG) scintigraphy and/or 24-h urine collection for catecholamines, metanephrines, and vanillylmandelic acid is advisable before FNA [32].

Some limitations to this study must be considered. The main shortcomings are the retrospective nature and the potential for bias in selecting patients who were referred for surgery. In this study, of the 89 patients diagnosed as pNEN by EUS-FNA, 40 patients (45 %) did not undergo surgery, so these patients were excluded from the study. These cases were inoperable and referred for chemotherapy or follow-up due to patient unwillingness to undergo surgery. In addition, patients in whom pNEN was suspected based on imaging, particularly when small in size (<10 mm) that could not be diagnosed by EUS-FNA, were followed up without surgery, and hence were excluded from our study. This may carry an unavoidable selection bias. Therefore, as shown in Table 4, the diagnostic performance of EUS-FNA was 100 % for pNENs <10 mm.

The negative-pressure suction techniques are also one of the important factors influencing the diagnostic performance. To date, few randomized, controlled trials have examined negative-pressure suction techniques. Puri et al. [33] concluded that the use of negative pressure did not improve diagnostic accuracy, but Kudo et al. [26] mentioned that a high negative-pressure suction technique is superior to normal negative-pressure suction in terms of obtaining sufficient material for histological diagnosis. The necessity for negative-pressure suction techniques remains controversial [34]. Consideration of this factor as a variable potentially affecting sampling adequacy would have been preferable, but the use of negative-pressure suction with a 10- or 20-mL syringe for almost all cases meant that such evaluation could not be performed.

As for needle size, Sakamoto et al. [35] reported that a 25G needle is less adequate for histological diagnosis compared with other needles, and Larghi et al. [6] argued that 19G is safe, feasible, and highly accurate for both diagnosis and Ki-67 determination. Needle size is thus an important factor affecting the accuracy of EUS-FNA for pNEN.

In 53 cases (excluding the five patients for whom both 22G and 25G needles were used), comparisons were made between 19G, 22G, and 25G needles, revealing no significant differences. However, the small number of cases makes reaching any firm conclusions difficult, and further studies are needed.

The strength of this study was that this is the first report to compare results of EUS-FNA with surgery as the gold standard, along with a complete description of diagnostic performance.

In conclusion, EUS-FNA offers a high accuracy for pNEN. However, tumor location in the pancreatic head and tumors with rich stromal fibrosis were associated with reduced sampling adequacy of EUS-FNA. We recommend the addition of T2-WI in all cases of suspected pNEN before EUS-FNA and use of a variety of complementary diagnostic modalities when the lesion appears iso- or hypointense on MRI.

## Electronic supplementary material

Below is the link to the electronic supplementary material.

Supplementary material 1 (PPT 583 kb) **Figure S3.** Imaging findings of paraganglioma. **A, B)** Axial and coronal images of CT show cystic degenerated tumor appearing to arise from the pancreatic parenchyma. **C, D)** EUS shows cystic degenerated tumor, punctured to the solid component. No hypersensitive surges were encountered during the procedure

Supplementary material 2 (PPT 777 kb) **Figure S4.** Microscopic findings of SPN. **A)** Diff-Qick stain of EUS-FNA cytology shows small, relatively round cells with wide cytoplasm. **B)** HE staining of a EUS-FNA cell block shows cells with mild atypia arranged in strands, with no findings of pseudopapillary pattern with fibrovascular stalks. **C)** HE staining of resected specimen shows pseudopapillary pattern with fibrovascular stalks as features of SPN
