# Supplementary figures and images for: Diagnostic performance and factors influencing the accuracy of EUS-FNA of pancreatic neuroendocrine neoplasms
Source: J Gastroenterol. 2016 Jan 14;51(9):923–30. doi: 10.1007/s00535-016-1164-6 (PMC4990623; doi:10.1007/s00535-016-1164-6)

## Slide 1
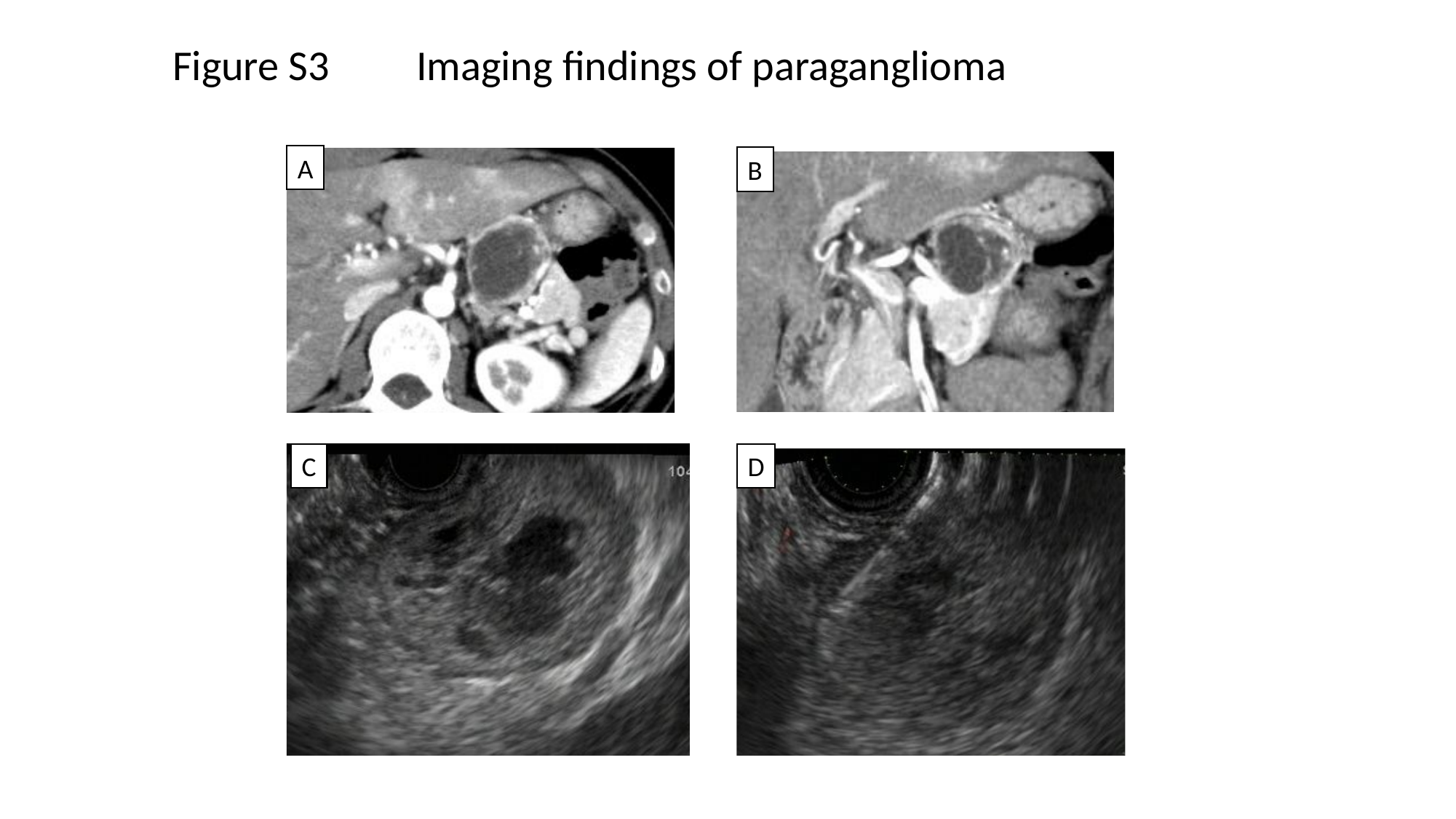

# Figure S3 Imaging findings of paraganglioma
A
B
D
C

Supplement: Supplementary file 1 — Supplementary material 1 (PPT 583 kb) Figure S3. Imaging findings of paraganglioma. A, B) Axial and coronal images of CT show cystic degenerated tumor appearing to arise from the pancreatic parenchyma. C, D) EUS shows cystic degenerated tumor, punctured to the solid component. No hypersensitive surges were encountered during the procedure [file 535_2016_1164_MOESM1_ESM.ppt]

## Slide 1
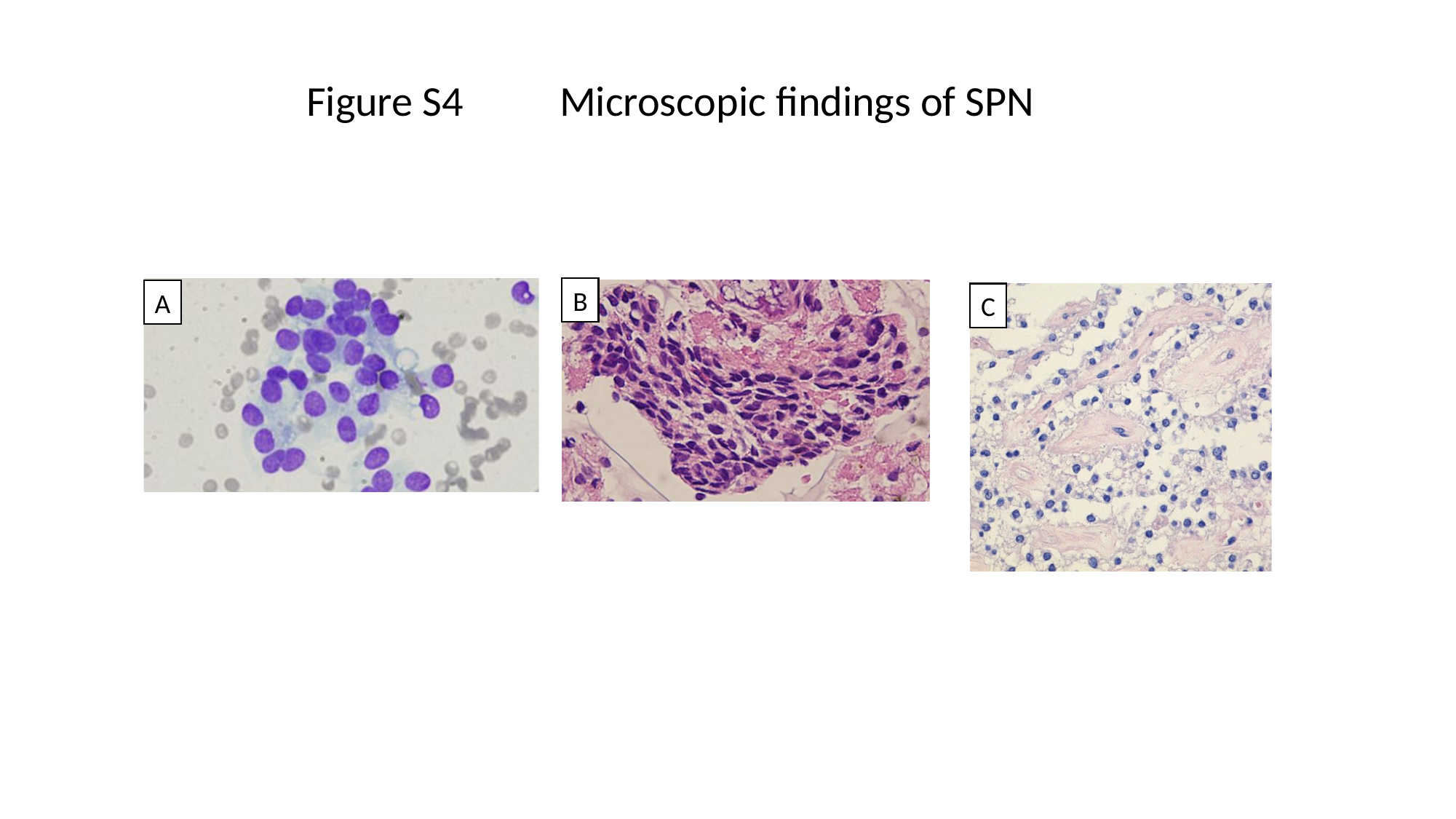

Figure S4 Microscopic findings of SPN
B
A
C

Supplement: Supplementary file 2 — Supplementary material 2 (PPT 777 kb) Figure S4. Microscopic findings of SPN. A) Diff-Qick stain of EUS-FNA cytology shows small, relatively round cells with wide cytoplasm. B) HE staining of a EUS-FNA cell block shows cells with mild atypia arranged in strands, with no findings of pseudopapillary pattern with fibrovascular stalks. C) HE staining of resected specimen shows pseudopapillary pattern with fibrovascular stalks as features of SPN [file 535_2016_1164_MOESM2_ESM.ppt]
